# Glycosaminoglycans as Tools to Decipher the Platelet Tumor Cell Interaction: A Focus on P-Selectin

**DOI:** 10.3390/molecules25051039

**Published:** 2020-02-26

**Authors:** Svenja Schwarz, Lukas Maria Gockel, Annamaria Naggi, Uri Barash, Martina Gobec, Gerd Bendas, Martin Schlesinger

**Affiliations:** 1 Pharmaceutical Institute, University of Bonn, An der Immenburg 4, 53121 Bonn, GermanyLukas.Gockel@uni-bonn.de (L.M.G.); gbendas@uni-bonn.de (G.B.); 2 G. Ronzoni Institute for Chemical and Biochemical Research, Via G. Colombo 81, 20133 Milan, Italy; naggi@ronzoni.it; 3 Cancer and Vascular Biology Research Center, Rappaport Faculty of Medicine, Technion, PO Box 9649, Haifa 31096, Israel; ubarash@technion.ac.il; 4 Faculty of Pharmacy, University of Ljubljana, Aškerčeva 7, SI-1000 Ljubljana, Slovenia; martina.gobec@ffa.uni-lj.si

**Keywords:** RO-heparin, 2-*O*-desulfated heparin, hexasaccharide heparin fragment, decasaccharide heparin fragment, unfractionated heparin, low molecular weight heparin, platelets, P-selectin, platelet aggregation, platelet secretion, tumor metastasis

## Abstract

Tumor cell–platelet interactions are regarded as an initial crucial step in hematogenous metastasis. Platelets protect tumor cells from immune surveillance in the blood, mediate vascular arrest, facilitate tumor extravasation, growth, and finally angiogenesis in the metastatic foci. Tumor cells aggregate platelets in the bloodstream by activation of the plasmatic coagulation cascade and by direct contact formation. Antimetastatic activities of unfractionated or low molecular weight heparin (UFH/LMWH) can undoubtedly be related to attenuated platelet activation, but molecular mechanisms and contribution of contact formation vs. coagulation remain to be elucidated. Using a set of non-anticoagulant heparin derivatives varying in size or degree of sulfation as compared with UFH, we provide insight into the relevance of contact formation for platelet activation. Light transmission aggregometry and ATP release assays confirmed that only those heparin derivatives with P-selectin blocking capacities were able to attenuate breast cancer cell-induced platelet activation, while pentasaccharide fondaparinux was without effects. Furthermore, a role of P-selectin in platelet activation and signaling could be confirmed by proteome profiler arrays detecting platelet kinases. In this study, we demonstrate that heparin blocks tumor cell-induced coagulation. Moreover, we identify platelet P-selectin, which obviously acts as molecular switch and controls aggregation and secretion of procoagulant platelets.

## 1. Introduction

Interaction of tumor cells with platelets is likely one of the most decisive steps in hematogenous cancer cell dissemination. Upon arrival in the blood, tumor cells activate platelets and, resulting thereof, are immediately encased by platelets [1]. Platelets confer a multitude of survival advantages to tumor cells and crucially contribute to successful metastatic nodule formation. Platelets protect tumor cells from shear stress and detrimental Natural killer cell assaults by downregulation of NK cell activating receptor NKG2D [2]. Platelets are able to induce an invasive epithelial to mesenchymal-like phenotype to breast cancer cells accompanied by an increased number of metastases in the lungs of mice [3]. The close interaction between platelets and tumor cells is mediated by platelet adhesion receptors such as P-selectin or GPIIb/IIIa among different others and enables adhesion of platelet tumor cell aggregates at the endothelium for a subsequent extravasation [4,5]. As a result of platelet activation and the coagulation status, cancer patients often reveal a three- to four-fold increased risk of venous thromboembolism as compared with healthy subjects [6]. 

Tumor cells utilize different stimulation pathways to initiate platelet activation in their close proximity. First, several tumor cells express tissue factor (TF) on their cell membranes, which activates the plasmatic coagulation cascade resulting in thrombin formation [7,8]. This is regarded as one of the main stimuli for platelet activation. Thrombin, in turn, induces human platelet activation via cleavage of the protease-activated receptor-1 (PAR-1) [8,9,10]. Second, cancer cell secretion of soluble mediators like ADP [11], or thromboxane A2 (TXA2) also has an impact on the status of platelet activation [12]. Notably, tumor cell-derived high-mobility group box 1 protein (HMGB1), which binds to toll-like receptor 4 (TLR4), has also been attributed to tumor cell-induced platelet aggregation [13]. A third line of evidence suggests that platelet activation is also driven by direct contact formation between platelets and tumor cells involving different platelet adhesion receptors [14]. Recently, platelet glycoprotein VI and integrin α6β1 were recognized to induce platelet activation upon binding to tumor cell expressing galectin-3 or ADAM-9, respectively [15,16]. A blockade of the platelet tumor cell communication could reduce tumor cell dissemination, and finally improve patients’ outcome. The glycosaminoglycan heparin, which is routinely administered to cancer patients to prevent venous thrombosis, is currently critically considered to prolong patients’ survival by attenuation of tumor metastasis [17,18]. A beneficial effect of low molecular weight heparin (LMWH) on cancer progression and patients’ survival has been observed in clinical trials [19,20], whereas other studies could not identify a significant survival benefit [21,22]. For heparin, various different mechanisms have been identified which interfere with the metastatic spread of tumor cells such as inhibition of adhesion receptors or enzymes such as heparanase [23,24]. 

Recently, we revealed that unfractionated heparin (UFH) blocks the tumor cell induced thrombin formation and concomitantly inhibits a platelet activation mediated by direct adhesion. In contrast, the pentasaccharide fondaparinux inhibited thrombin generation but failed to reduce platelet activation induced by direct binding [10]. However, the platelet receptors responsible for the observed heparin inhibition remained elusive. In this study, we attempted to resolve these questions with the use of different non-anticoagulant heparin derivatives varying in size, degree of sulfation and oxidation as research tools. We identified P-selectin as the adhesion receptor blocked by heparin. Moreover, we revealed that P-selectin is involved in platelet granule secretion and has an impact on platelet intracellular signaling. These results indicate that P-selectin has a broader implication in platelets than a simple adhesive function.

## 2. Results

### 2.1. Breast Cancer Cell-Induced Platelet Activation and Secretion

**Figure 1:**
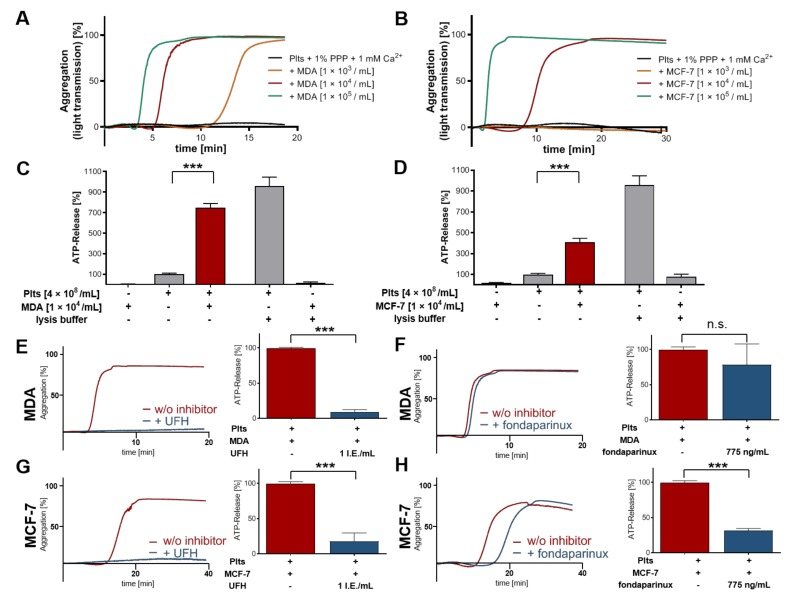
Tumor cell-induced platelet secretion and aggregation. (**A**,**B**) Representative traces showing platelet-tumor cell aggregation in response to increasing concentrations of (A) MDA-MB-231 cells for 20 min (*n* = 5) or (B) MCF-7 cells for 30 min; (**C**) quantification of ATP release from resting platelets, 1 × 10^4^ MDA-MB-231 cells/mL, platelets co-incubated with 1 × 10^4^ MDA-MB-231 cells/mL for 30 min, platelets treated with 1% Triton X-100, or MDA-MB-231 cells (1 × 10^4^ cells/mL) treated with 1% Triton X-100, respectively; (**D**) quantification of ATP release from resting platelets, MCF-7 cells, platelets co-incubated with MCF-7 cells for 30 min, platelets treated with 1% Triton X-100, or MCF-7 cells (1 × 10^4^ cells/mL) treated with 1% Triton X-100, respectively; (**E**) representative traces showing platelet aggregation in response to MDA-MB-231 cells (1 × 10^4^/mL) or MCF-7 cells (1 × 10^4^/mL) (*n* = 5). Platelets were preincubated with 1 IU/mL UFH (left part of the figure) (*n* = 5). Quantification of ATP release from MDA-MB-231 (1 × 10^4^/mL) cell or MCF-7 (1 × 10^4^/mL) cells stimulated platelets preincubated with 1 IU/mL UFH (*n* = 5) (right part of the figure); (**F**) representative traces showing platelet aggregation in response to MDA-MB-231 cells (1 × 10^4^/mL) or MCF-7 cells (1 × 10^4^/mL) (*n* = 5). Platelets were preincubated with 775 ng/mL fondaparinux (left part of the figure) (*n* = 5). Quantification of ATP release from MDA-MB-231 (1 × 10^4^/mL) cell or MCF-7 cells (1 × 10^4^/mL) stimulated platelets preincubated with 775 ng/mL fondaparinux (*n* = 5) (right part of the figure). *** *p* < 0.001 indicated statistical significance.

Different concentrations of highly aggressive MDA-MB-231 breast cancer cells were able to aggregate platelets in buffer by direct contact formation (Figure 1A). The slightly lower capacity for platelet activation of the low metastatic MCF-7 breast cancer cell line became evident by the longer time frame until platelet aggregation, and the fact that the lowest cell density (1 × 10^3^ MCF-7 cells/mL) completely failed to initiate platelet aggregate formation (Figure 1B). Next, we focused on tumor cell-induced platelet dense granules secretion, therefore, 1 × 10^4^ MDA-MB-231 or 1 × 10^4^ MCF-7 cells/mL were added to platelets in buffer, and ATP release was quantified with a luciferin-based ATP-determination kit. Additionally, we evaluated whether non-activated platelets, MDA-MB-231, or MCF-7 cells secrete significant amounts of ATP by themselves. While coincubation of platelets with MDA-MB-231 cells for 30 min induced a seven-fold increase in ATP levels as compared with unstimulated platelets (Figure 1C), MDA-MB-231 cells alone revealed hardly any ATP secretion. For MCF-7 cells, ATP release from platelets granules was elevated four-fold by activation of platelets after 30 min of co-incubation (Figure 1D). Thus, both tumor cell lines are potent platelet activators, nonetheless, MBA-MB-231 cells exhibit a more intense platelet secretion capacity as compared with MCF-7 cells. The observed release of ATP is attributed to the secretion from activated platelets and not to the intracellular levels in tumor cells. Namely, if MBA-MB-231 or MCF-7 cells were lysed with 1% Triton X-100, only a minor or no increase in ATP could be observed (Figure 1C,D). Next, we addressed the potential impact of UFH on tumor cell-induced platelet activation. Treatment of platelets with 1 IU/mL UFH prior to tumor cell addition completely blocked the MDA-MB-231 or MCF-7 cell induced aggregation, as well as ATP release (Figure 1E). In contrast, pentasaccharide fondaparinux had no impact on platelet aggregation or ATP release induced by MDA-MB-231 cells. For MCF-7 cells, platelet aggregation was slightly delayed and ATP release was reduced (Figure 1F).

### 2.2. Inhibition of Platelet Aggregation and Dense Granule Secretion by Modified Heparin Derivatives

**Figure 2:**
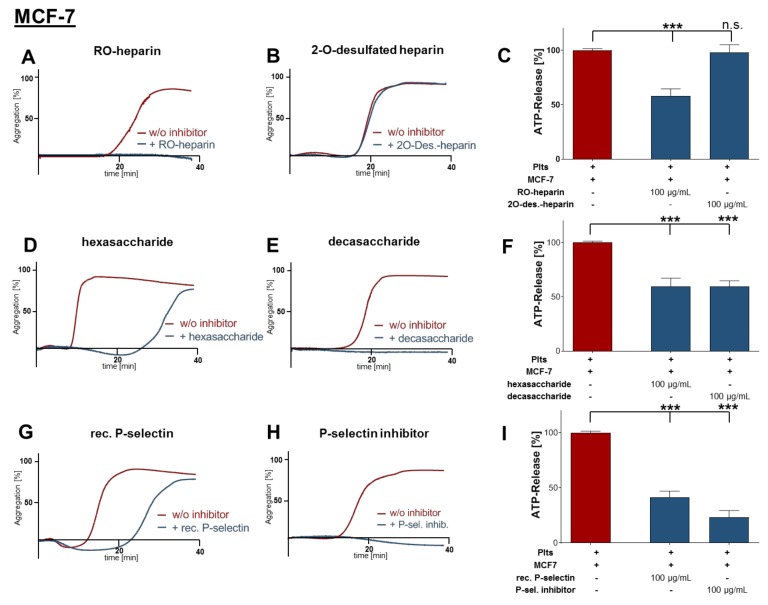
Impact of heparin derivatives on platelet aggregation and secretion. (**A**,**B**) Representative traces showing platelet-tumor cell aggregation in response to MCF-7 cells. Platelets were preincubated for 30 min with (**A**) 100 µg/mL RO-heparin or (**B**) 100 µg/mL 2-*O*-desulfated heparin; (**C**) quantification of ATP release from MCF-7 cell stimulated platelets preincubated with 100 µg/mL RO-heparin or 100 µg/mL 2-*O*-desulfated heparin; (**D**,**E**) representative traces showing platelet-tumor cell aggregation in response to MCF-7 cells, platelets were preincubated with (**D**) 100 µg/mL hexasaccharide or (**E**) 100 µg/mL decasaccharide (*n* = 5); (**F**) quantification of ATP release from MCF-7 cell stimulated platelets preincubated with 100 µg/mL hexasaccharide or 100 µg/mL decasaccharide; (**G**) representative traces showing platelet-tumor cell aggregation in response to MCF-7 cells. Tumor cells were preincubated with 1 µg/1000 cells recombinant human P-selectin (*n* = 5); (**H**) representative traces showing platelet-tumor cell aggregation in response to MCF-7 cells. Platelets were preincubated with 100 µg/mL P-selectin inhibitor (*n* = 5); (**I**) quantification of ATP release from MCF-7 cell (preincubated with 1 µg recombinant human P-selectin/1000 cells in some experiments) stimulated platelets preincubated with 100 µg/mL P-selectin inhibitor. *** *p* < 0.001 indicated statistical significance.

To determine the platelet receptor which is blocked by heparin and responsible for platelet activation by direct tumor cell contact, different non-anticoagulant heparin derivatives were utilized. First, platelets were preincubated with reduced oxyheparin (RO-heparin). MCF-7 cell mediated aggregation was completely blocked by RO-heparin (Figure 2A). RO-heparin is a non-anticoagulant heparin derivative with high P-selectin inhibitory activity [25]. Next, we applied 2-*O*-desulfated heparin which possessed absolutely no effect on MCF-7 cell induced aggregation (Figure 2B); 2-*O*-desulfated heparin is non-anticoagulant and has only a very weak binding affinity towards P-selectin. In close accordance, RO-heparin also decreased MCF-7 cell induced ATP release for 70%, whereas 2-*O*-desulfated heparin did not depress ATP secretion from platelets (Figure 2C). In order to evaluate P-selectin as potential heparin target in the course of platelet tumor cell communication, we applied hexa- and decasaccharide heparin fragments. For this approach, it is commonly agreed that at least six heparin saccharide units are required for a potent P-selectin inhibition [26]. This is the reason for the inability of fondaparinux to interfere in the tumor cell platelet communication. Additionally, fondparinux contains solely one 2-*O*-sulfo-α-l-Iduronic acid, a saccharide unit which is obviously relevant for P-selectin inhibition. 

Hexasaccharide fragment delayed MCF-7 induced platelet aggregation, whereas decasaccharide fragment completely diminished aggregation (Figure 2D,E). Both fragments also attenuated ATP secretion to a similar level (Figure 2F). Notably, these findings as compared with the inability of fondaparinux, as a known inactive compound on P-selectin [27], further emphasize this adhesion receptor as a probable target in this context. To further confirm the role of platelet P-selectin, recombinant human P-selectin was added aiming to interfere with tumor cell–platelet interaction. We noted that recombinant P-selectin only had a minor effect on platelet aggregation (Figure 2G). In contrast, the presence of a specific P-selectin small molecule inhibitor (bimosiamose), which has previously been clinically evaluated [28], blocked platelet cell aggregation completely (Figure 2H). In corresponding ATP release assays, recombinant human P-selectin reduced ATP levels by 59% and specific P-selectin inhibitor by 77%, respectively (Figure 2I).

In a comparable set of experimental approaches using the respective heparin derivatives, the role of P-selectin could also be confirmed for MDA-MB-231 cells activating platelets (Figure S1). 

### 2.3. Heparin Mediated P-Selectin Blockade Subsequent to Tumor Cell Interaction

**Figure 3:**
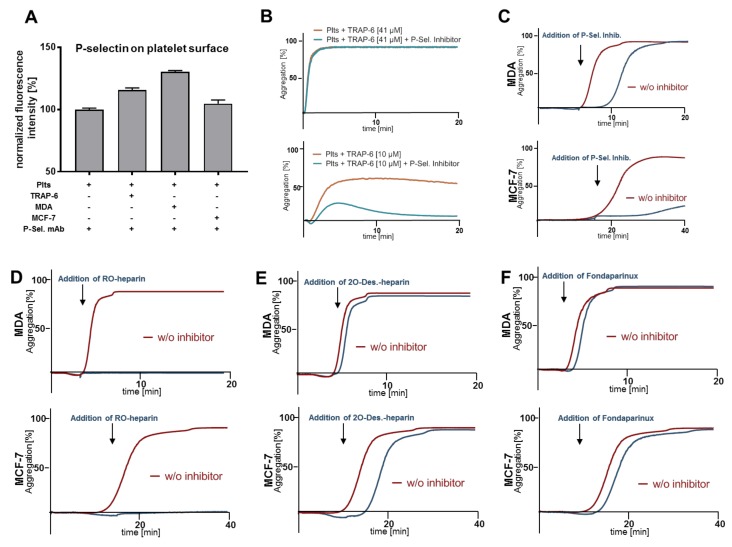
Impact of heparin derivatives on platelet aggregation and secretion after platelet shape change. (**A**) Platelets were stimulated with TRAP-6, MDA-MB-231, or MCF-7 cells, respectively, and P-selectin expression was determined by flow cytometry; (**B**) representative traces showing platelet-tumor cell aggregation in response to PAR-1 receptor agonist TRAP-6 (41 µM or 10 µM, respectively). Platelets were preincubated for 30 min with 100 µg/mL P-selectin inhibitor (*n* = 5); (**C**) representative traces showing platelet-tumor cell aggregation in response to MDA-MB-231 or MCF-7 cells, respectively. P-selectin inhibitor (100 µg/mL) was added 5 min (10 min in case of MCF-7 cells) after tumor cell addition when platelet shape change was in progress (*n* = 5); (**D**–**F**) representative traces showing platelet-tumor cell aggregation in response to MDA-MB-231, or MCF-7 cells, respectively, when RO-heparin (100 µg/mL) (**D**), 2-*O*-desulfated heparin (100 µg/mL) (**E**), or fondaparinux (775 ng/mL) (**F**) were added 5 min/10 min after tumor cell addition when platelet shape change was in progress (*n* = 5).

P-selectin is expressed at a basal level on resting platelets. After platelet activation, the majority of P-selectin molecules located in α-granules are translocated to the platelet membrane. By flow cytometry, we detected P-selectin on platelet membranes. Their number was increased after activation either with PAR-1 receptor activating peptide TRAP-6, or direct contact with tumor cells (MDA-MB-231 or MCF-7 cells) (Figure 3A). In order to test whether P-selectin is involved in physiological platelet activation by PAR-1 ligation, platelets preincubated with P-selectin inhibitor were stimulated either with high or low concentrations of TRAP-6 before aggregation was monitored (Figure 3B). High TRAP-6 concentrations induced complete platelet aggregation and addition of the P-selectin inhibitor could not interrupt this process. In contrast, platelets stimulated with low TRAP-6 concentrations revealed a 50% aggregation which was susceptible to the P-selectin inhibitor (Figure 3B, lower part). Thus, P-selectin activating signals seem to be implicated in an ongoing physiological platelet activation process. 

Next, we explored whether P-selectin, which appears on the platelet membrane after initial contact and activation by tumor cells, is also involved in the process of activation and aggregation. Thus, platelets were stimulated either with MDA-MB-231 or MCF-7 cells, respectively, and P-selectin inhibitor was added afterwards when platelets´ shape change was already in progress (indicated with arrows in Figure 3C). The late application of the P-selectin inhibitor retarded aggregation in the case of MDA-MB-231 cells, and completely blocked MCF-7 induced aggregation (Figure 3C). Late addition of RO-heparin abrogated aggregation for both tumor cell lines (Figure 3D). In contrast, late administration of 2-*O*-desulfated heparin had no impact on MDA-MB-231 and merely a slight impact on MCF-7 cell induced aggregation (Figure 3E). Finally, when fondaparinux was added at the moment of platelet shape change, no inhibitory effect on tumor cell induced aggregation could be observed (Figure 3F). 

### 2.4. Impact of P-Selectin Inhibition on Platelet α-Granule Secretion

**Figure 4:**
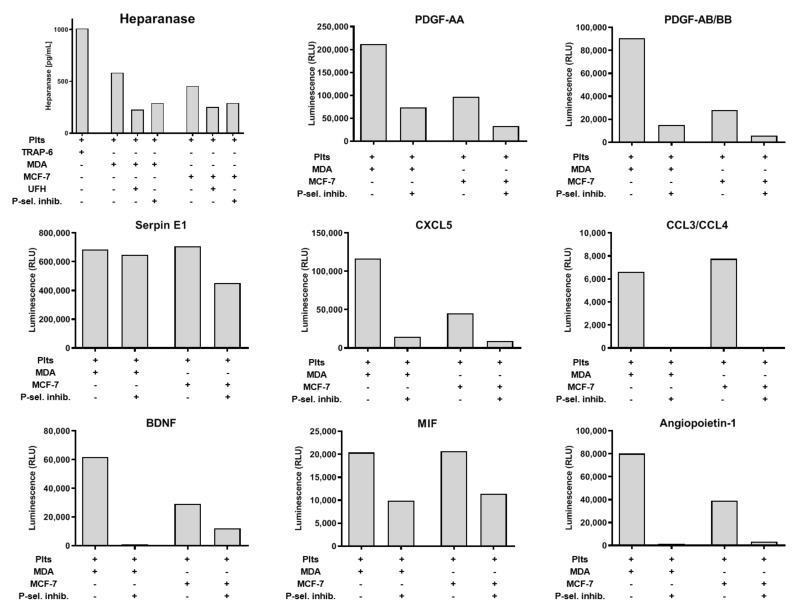
P-selectin inhibition impacts platelets’ secretion. Heparanase secretion from platelets activated with TRAP-6, MDA-MB-231 cells, or MCF-7 cells, respectively. Platelets were preincubated with 1 IU/mL UFH or 100 µg/mL P-selectin inhibitor, for 30 min in some cases. Proteome profiler mediated quantification of the platelet secretion due to MDA-MB-231 or MCF-7 cell (1 × 10^4^ cells/mL) induced secretion. Platelets were preincubated with P-selectin inhibitor for 30 min, as indicated in the figure, and PDGF-AA, PDGF-AB/BB, Serpin E1, CXCL5, CCL3/CCL4, BDNF, MIF, and angiopoietin were quantified in the releasates (*n* = 1).

Since we have focused on aggregation and dense granules release, we sought to determine the impact of P-selectin on α-granules release. First, we quantified the release of heparanase from platelets after MCF-7 or MDA-MB-231 co-incubation. Heparanase is involved in many pathologic situations and increased heparanase concentrations contribute to tumorigenicity and metastasis [29]. PAR-1 activation with TRAP-6 induced a more pronounced heparanase release than platelet activation by the two tumor cell lines. Nevertheless, the heparanase secretion release from platelets induced by both cell lines was sensitive to P-selectin inhibition by either UFH or P-selectin inhibitor (Figure 4). To gain a deeper insight into platelets’ secretion and its modulation by P-selectin inhibition, we applied proteome profiler arrays to quantify the release of eight molecules stored in platelets’ α-granules subsequent to MDA-MB-231 or MCF-7 activation. In detail, we quantified Platelet-derived growth factor (PDGF)-AAPDGF-AA, PDGF-AB/BB, Plasminogen activator inhibitor-1 (serpin E1), chemokines CXCL5, CCL3/CCL4, Brain-derived neurotrophic factor (BDNF), Macrophage migration inhibitory factor (MIF), and angiopoietin. For all these molecules, MDA-MB-231 cells induced a stronger granule release as compared with MCF-7 cells (Figure 4). Interestingly, release of some molecules (e.g., serpin E1) was only slightly affected by P-selectin inhibition. In contrast, secretion of other molecules such as CCL3/CCL4 or angiopoietin was completely blocked by P-selectin inhibition.

### 2.5. P-Selectin Signaling in Platelets

**Figure 5:**
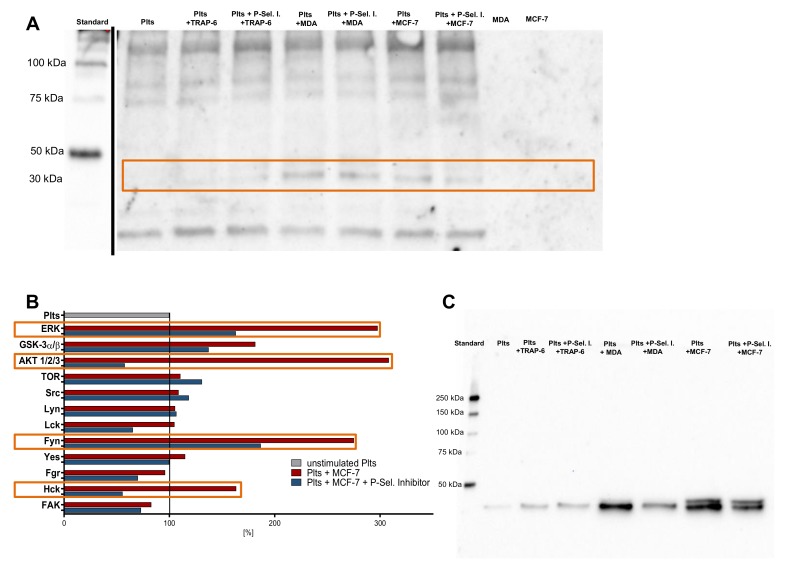
P-selectin mediated signaling in platelets. (**A**) Immunoblot analysis using an anti-phosphoserine/phosphothreonine/phosphotyrosine antibody of lysates from resting platelets, platelets after activation with 41 µM TRAP-6, after co-incubation with MDA-MB-231 or MCF-7 cells (1 × 10^4^ cells/mL), respectively. Platelets were preincubated with 100 µg/mL P-selectin inhibitor for 30 min in some samples. Standard from the same gel at an earlier time was inserted in the figure to avoid overexposure. The orange box indicates changes in platelet phosphoproteome; (**B**) proteome profiler Phospho-Kinase array kit of lysates from platelets co-incubated with P-selectin inhibitor or without coincubation and stimulated with MCF-7 cells. Twelve kinases (as indicated in the figure) involved in platelet signaling were quantified simultaneously. The orange boxes indicate changes in phosphorylation of different kinases due to P-selectin inhibition; (**C**) immunoblot analysis of lysates using an anti-pERK1/2 antibody from resting platelets, platelets after activation with 41 µM TRAP-6, after coincubation with MDA-MB-231, or MCF-7 cells (1 × 10^4^ cells/mL), respectively. Platelets were preincubated with 100 µg/mL P-selectin inhibitor in some samples.

To evaluate whether P-selectin initiates a signaling cascade in platelets, we first analyzed the platelet status of phosphotyrosine, phosphothreonine, and phosphoserine. We detected changes in platelets phosphoproteome due to activation with TRAP-6, MDA-MB-231, or MCF-7 cells, as well as when preincubation of P-selectin inhibitor was present (Figure 5A, indicated with the orange box). Proteins with a molecular mass of approximately 35 kDa exhibit an increased phosphorylation as compared with non-activated platelets. To investigate which platelet kinases were involved in P-selectin signaling pathways, we applied a proteome profiler Phospho-Kinase array kit focusing in parallel on 12 kinases present in platelets. Kinases of unstimulated platelets were used as references and set to 100%. Src-family kinase Fyn and Hck, protein kinase B (Akt) and extracellular signal-regulated kinase (Erk) revealed increased phosphorylation after tumor cell activation as compared with non-stimulated platelets. Application of P-selectin inhibitor significantly reduced phosphorylation of these kinases (Figure 5B, indicated with the orange boxes). To confirm the proteome profiler Phospho-Kinase array results, we conducted Western blots and found an increased phosphorylation of Erk after platelet tumor cell contact (Figure 5C). Preincubation of platelets with P-selectin inhibitor strongly reduced Erk phosphorylation, whereas total Erk expression was minimally affected (data not shown). Hence, we propose that P-selectin induces signaling in platelets which likely involves Fyn, Hck, Akt, and Erk, which finally culminates in granules secretion and aggregation.

## 3. Discussion

Tumor cell platelet interaction is regarded as a key event in the process of hematogenous metastasis. Upon activation, platelets secrete various protumorigenic biomolecules that propagate metastasis and vascularization of metastatic foci [30,31]. In general, tumor cells utilize three different mechanisms to induce platelet activation. In addition to the secretion of molecules such as ADP or TXA2 [32], and activation of the plasmatic coagulation cascade [33], juxtacrine interaction is the third and highly relevant pathway that contributes to platelet activation [16,34,35,36]. 

In the present study, we make use of different glycosaminoglycan derivatives (RO-heparin, 2-*O*-desulfated heparin, hexasaccharide and decasaccharide heparin fragments, and UFH) as tools to delineate that the platelet adhesion molecule P-selectin is responsible for the interaction between platelets and tumor cells [10]. P-selectin mediates platelet aggregation and secretion of platelets α- and dense granules, subsequent to tumor cell contact. The role of P-selectin in cancer metastasis is well known for decades since P-selectin initiates the interaction of platelets with sialylated fucosylated mucins on circulating tumor cells [37,38]. However, P-selectin was regarded as a simple adhesion receptor with no signaling function. Here, we demonstrate that P-selectin participates, at least partially, in platelet activation and expedites different intracellular signaling events culminating in platelet aggregation and secretion. Our data support the notion that UFH potentially has antimetastatic effects through mitigation of the platelet response towards cancer cells. 

LMWH is currently the state-of-the-art drug for cancer patients in order to prevent venous thromboembolism (VTE) and cardiovascular ischemic events since cancer patients have an increased risk to suffer from VTE as compared with healthy individuals [17]. For heparin, a lot of tumor-associated targets beyond anticoagulation have been identified in the last years. For instance, heparin efficiently blocks enzymes such as heparanase or matrix metalloproteinases, binds growth factors and chemokines, blocks various adhesion receptors, and can sensitize tumor cells for cytotoxic drugs among many other mechanisms [39,40,41]. These effects were reflected in some clinical trials which exhibited an increased survival for heparin treated cancer patients [19,20]. Nonetheless, several recent clinical trials could not validate a potential survival benefit for heparinized patients [21,22]. Furthermore, direct oral anticoagulants (DOACs) are currently investigated for VTE prevention in cancer patients as compared with LMWHs. Initial evidence suggests that patients receiving DOACs have a reduction in recurrent VTE with an increased risk of major or clinically relevant non-major bleedings as compared with LMWH [42,43,44,45]. Nonetheless, for some cancer patient populations, classified according to cancer entity, stage of disease, or expression patterns of distinct molecules or receptors among many other factors, LMWH treatment could potentially bear some beneficial antitumorigenic effects since DOACs do not address other tumor relevant targets [18].

Several lines of evidence support our results of P-selectin as an activating platelet receptor. First, P-selectin-deficient mice show a 40% increased bleeding time as compared with wild-type mice on amputation of the tail tip [46]. This corresponds with our data of delayed aggregation due to P-selectin blockade. The secretion machinery is also affected in P-selectin deficient mice, since lower levels of vascular endothelial growth factor (VEGF) were detectable in the supernatant of co-culture experiments with B16F10 melanoma cells [47]. This is also in line with our observations of reduced or impeded granule secretion upon P-selectin blockade. A vast amount of knowledge dealing with the role of P-selectin in metastasis is available and a blockade of P-selectin culminated in tremendous reduction of metastatic nodules for instance of colon cancer cells in the lungs of mice [48,49]. In most of these studies, P-selectin has been regarded as an adhesion receptor that solely mediates the close contact between tumor cells and platelets conferring survival stimuli to tumor cells. However, more recently, the paradigm of P-selectin biology has been amplified beyond a basic adhesive function.

Théorêt and colleagues revealed that the interaction of P-selectin with its high-affinity ligand, a recombinant soluble form of P-selectin glycoprotein ligand-1 (rPSGL-1), enhanced platelet activation, adhesion, and microaggregate formation [50]. Thrombus formation and microaggregates were both enhanced by rPSGL-1 in wild-type, but not in CD62P^−/−^ mice. Furthermore, Merten et al. exhibited that sulfated glycosphingolipids sulfatides aggregated washed platelets in a dose-dependent manner and enhanced platelet aggregation in platelet-rich plasma due to P-selectin binding [51]. Sathish induced calcium entry in human platelets by antibody crosslinking of human P-selectin [52]. In addition, histidine, serine, and threonine phosphorylation of P-selectin cytoplasmic residues was demonstrated due to stimulation of platelets with thrombin, phorbol 12-myristate 13-acetate, or collagen, respectively [53,54].

Nolo et al. demonstrated a P-selectin dependent neuroblastoma growth. These studies referred to a P-selectin induced tumor cell signaling by binding to tumor cell expressed counter-receptors [55], which we further emphasize here. By an analysis of the phosphorylation profile of kinases, we illustrate that P-selectin is involved in platelet signaling via phosphorylation of Src family kinases Fyn and Hck, protein kinase B (Akt), and finally Erk which appears to be a prerequisite for platelet granules secretion and ultimately aggregation [56,57,58]. 

P-selectin mediated signaling in platelets is hardly investigated since P-selectin was regarded as a simple adhesion receptor located in α-granules with less signaling function [59]. Nonetheless, our findings are corroborated by Becker et al. who recently showed that P-selectin and p38 MAPK signaling are involved in secretion of acid sphingomyelinase from platelets after melanoma cell interaction. In turn, acid sphingomyelinase increased pulmonary metastasis and contributed to platelet melanoma cell interaction [60]. A previous study by Matsuo et al. also provided a link between P-selectin on platelets and p38 MAPK expression, since p38 MAPK knockout mice expressed less P-selectin mRNA, as well as P-selectin protein in platelets. Additionally, the p38 MAPK knockout mice exhibited a reduced number of metastases in the lungs after tumor cell injection [61]. Earlier reports from Esumi et al. and Bradley et al. revealed that the first stage of platelet activation by tumor cells depended on the expression of glycoproteins or glycolipids with functionally important sialic acid and N-linked carbohydrate residues. These data clearly point to a P-selectin involvement in platelet activation since glycoproteins and glycolipids are P-selectin ligands. However, the exact mechanism of how glycoproteins and glycolipids initiated platelet activation remained elusive [62,63]. More recently, Wang et al. revealed a P-selectin interaction with intracellular talin-1, which subsequently activated integrin GPIIb/IIIa and resulted in a P-selectin-GPIIb/IIIa-talin complex, and finally platelet accumulation in tumor tissue [64]. These results indicated a P-selectin signaling and crosstalk with other adhesion receptors. Our data are in agreement with Battinelli et al. who found that preincubation of platelets with heparin led to a shift in platelet secretion. Platelets exposed to heparin secreted significantly lower amounts of VEGF in response to MCF-7 mediated activation and the releasate comprised reduced angiogenic potential [65]. Thus, it is tempting to speculate that the observed effects are at least partially induced by a heparin-mediated blockade of P-selectin with a subsequent impact on platelet release characteristics, since we also detected a distinct change in platelets´ secretion by selective P-selectin inhibition. Moreover, it was previously shown that platelets contain different α-granules with different release characteristics [66]. Our data suggest that some molecules are apparently colocalized with P-selectin in the same α-granules. These α-granules have partially been released by means of tumor cell preactivation and the impact of P-selectin inhibition is rather low. Other α-granule subtypes have not yet been released from platelets after tumor cell stimulation and their secretion can be efficiently blocked by platelet P-selectin inhibition.

In summary, our data suggest that heparin and molecules with a distinct glycosaminoglycan structure efficiently interfere with the direct contact mediated platelet aggregation and secretion. Since LMWH has the ability to bind P-selectin and to simultaneously inhibit the plasmatic coagulation cascade, it is potentially a valuable drug for medication of certain cancer patients. 

## 4. Materials and Methods 

### 4.1. Glycosaminogylcans and Sulfated Polysaccharides

Unfractionated heparin (25,000 IU/mL) was purchased from Ratiopharm, Ulm, Germany. Non-anticoagulant reduced oxyheparin “RO-heparin” (“glycol-split” heparin) and 2-*O*-desulfated heparin (final concentration 100 µg/mL, respectively) were prepared as previously described [67]. RO derivatives are a form of “glycol-split” heparins and the C(2)-C(3) bonds of all nonsulfated uronic acid residues are cleaved, with maintenance of the original chain length. These derivatives have flexible joints along the polysaccharide chains and exhibit a high P-selectin binding and blocking capacity [25]. In contrast, 2-*O*-desulfated heparin has hardly any P-selectin blocking ability [25]. Chemically modified heparins had an average molecular weight in the order of 15 kD. All samples were recovered after freeze-drying and diluted to a concentration of 1 mg/mL. Hexa-, and decasaccharide fractions of dalteparin were isolated by size exclusion chromatography [68]. 

### 4.2. Cell Lines

Human breast cancer cell lines MDA-MB-231 and MCF-7 were grown in Dulbecco’s modified Eagle’s medium (DMEM) (Sigma-Aldrich, St. Louis, USA) with 10% (*v*/*v*) FCS, 1% l-glutamine, 100 U/mL penicillin, and 100 µg/mL streptomycin (plus 1% sodium pyruvate in case of MDA-MB-231). Cells were incubated at 37 °C in a humidified atmosphere containing 5% CO_2_. Cells were detached at 90% confluence using a solution of EDTA (0.2 g/L EDTA × 4 Na) for 10 min at 37 °C. All reagents were from Thermo Fisher Scientific Inc. (Waltham, MA, USA). Cell identity was evaluated using a STR profile analysis.

### 4.3. Preparation of Washed Platelets

Platelet-rich plasma was obtained from Institute for Experimental Hematology and Transfusion Medicine, University of Bonn, Medical Centre, in accordance to the declaration of Helsinki. Isolated human platelets (Plts) in buffer were prepared from platelet-rich plasma by centrifugation (670 g, 10 min, 22 °C) and resuspension (400 × 10^6^ Plts/mL) in recalcified (1 mM) platelet buffer (10 mM HEPES, 137 mM NaCl, 2.6 mM KCl, 1 mM MgCl_2_, 13.8 mM NaHCO_3_, 0.36 mM NaH_2_PO_4_, 5.5 mM d-glucose). Before use, 1% platelet-poor plasma was added. Prior to activation, in some experiments, platelets were preincubated with indicated heparin derivatives, or bimosiamose (TBC-1269; 1,6-bis(3-(3-carboxymethylphenyl)-4-(2-alpha-d-mannopyranosyloxy)phenyl)hexane) a selective selectin inhibitor (100 µg/mL; formerly Revotar Biopharmaceuticals, Hennigsdorf, Germany) for 30 min. In some experiments, tumor cells were preincubated with recombinant P-selectin (BioTechne, Wiesbaden, Germany).

### 4.4. Platelet Dense Granule Secretion Assay

Plts were coincubated with inhibitors or phosphate-buffered saline (PBS) for 30 min. Tumor cells were detached with EDTA and resuspended in PBS. Platelets (400 × 10^6^ Plts/mL) were activated with 1 × 10^4^ tumor cells/mL for 30 min. Platelets ATP secretion from dense granules was assessed by luminescence measurement using a luciferin-based ATP-Determination Kit (Thermo Fisher Scientific, Waltham, USA) and a FLUOstar Optima plate reader (BMG Labtech, Ortenberg, Germany). In some experiments, platelets (400 × 10^6^/mL) or tumor cells (1 × 10^4^/mL) were lysed with 1% Triton X-100 in PBS and ATP was quantified, subsequently.

### 4.5. Light Transmission Aggregometry

Measurement of tumor cell-induced platelet aggregation was performed by light transmission aggregometry using an APACT-4004 aggregometer (Haemochrom Diagnostica, Essen, Germany). Platelets were prepared and co-incubated with different inhibitors as described before. Platelets aggregation was induced by 1 × 10^4^ tumor cells/mL at 37 °C in adequate cuvettes, stirred continuously at 1000 rpm. Aggregate formation was measured by light transmission, with buffer set as 100% and platelets in buffer as 0% reference values. 

### 4.6. Western Blot 

Platelets were prepared, incubated with P-selectin inhibitor and activated with tumor cells or thrombin-receptor activating peptide 6 (TRAP-6) (41 µM, BioTechne) as described before. After activation, platelets were centrifuged (670 g, 10 min, 22 °C) and lysed with cell extraction buffer (Thermo Fisher), supplemented with 0.1 mM PMSF and protease inhibitors (1 μg/mL aprotinin, 1 μg/mL leupeptin) (Life Technologies, Carlsbad, CA, USA), according to manufacturer’s instructions. Lysate quantification, SDS-Page, and Western blots were performed using stain-free gels as described before [69]. Membranes were incubated with anti-phosphoserine/phosphothreonine/phosphotyrosine antibodies (antibodies-online GmbH, Aachen, Germany), or anti-pERK1/2 mAb (cell signaling technology, Danvers, USA), and anti-mouse IgG HRP-conjugated mAbs (Santa Cruz Biotechnology), respectively.

### 4.7. Cytokine and Phospho-Kinase Arrays

Membrane-based proteome profiler human XL cytokine and human phospho-kinase array kits (BioTechne) were used according to manufacturer’s protocol. Briefly, 400 × 10^6^ Plts/mL were activated with 1 × 10^4^ tumor cells for 30 min with or without inhibitor and centrifuged at 670 g. For human XL cytokine array, 200 µL of supernatant were added per membrane for 12 h. For human phospho-kinase array, 500 µg protein of the pellet were applied per membrane and incubated for 12 h. Pixel density analysis was performed with Image Lab software (Bio-Rad Laboratories, Munich, Germany).

### 4.8. Heparanase ELISA 

Heparanase release from platelets subsequent to tumor cell interaction or TRAP-6 activation was quantified with heparanase specific ELISA performed as described before [70].

### 4.9. Flow Cytometry

To determine P-selectin on human platelets, 1 µg FITC-labeled anti-P-selectin mAb or an appropriate mouse IgG1 kappa isotype control mAb (Thermo Fisher Scientific) were added to 4 × 10^7^ platelets. Platelet activation was induced by adding 41 µM TRAP-6, 1 × 10^3^ MDA-MB-231, or MCF-7 cells for 30 min, respectively. For each sample 1 × 10^4^ platelets were analyzed with a Guava easy Cyte 3 HT reader (Merck Millipore, Billerica, USA) after washing with PBS twice.

### 4.10. Statistical Analysis

Comparisons were performed using the software Prism™ (GraphPad Software, San Diego, CA, USA). Student’s t test was used to compare two groups, and one-way analysis of variance (ANOVA) was used for three or more groups. * *p* < 0.05, ** *p* < 0.01, and *** *p* < 0.001 indicated statistical significance.

## 5. Conclusions

Utilizing the known structure-activity relationships of the heparin glycosaminoglycan (GAG) structure, to balance anticoagulant and anti-adhesive properties, we provide evidence that P-selectin has an adhesive function in platelets and additionally initiates an intracellular signaling that contributes to α- and dense granule secretion and aggregation. Glycosaminoglycans, for instance LMWH, that block the plasmatic coagulation cascade and interfere with P-selectin potentially provide an advantage for inhibition of tumor cell induced platelet activation as compared with DOACs. Thus, for prevention and treatment of VTE in cancer patients, the substitution of LMWHs with DOACs should be critically evaluated in further studies.

## Supplementary Materials

The following are available online at https://www.mdpi.com/1420-3049/25/5/1039/s1, Figure S1: Impact of heparin derivatives on platelet aggregation and secretion.
