# Supplementary material for: Glycosaminoglycans as Tools to Decipher the Platelet Tumor Cell Interaction: A Focus on P-Selectin
Source: Molecules. 2020 Feb 26;25(5):1039. doi: 10.3390/molecules25051039 (PMC7179249; doi:10.3390/molecules25051039)
Supplement: Supplementary file 1 [file molecules-25-01039-s001.pdf]

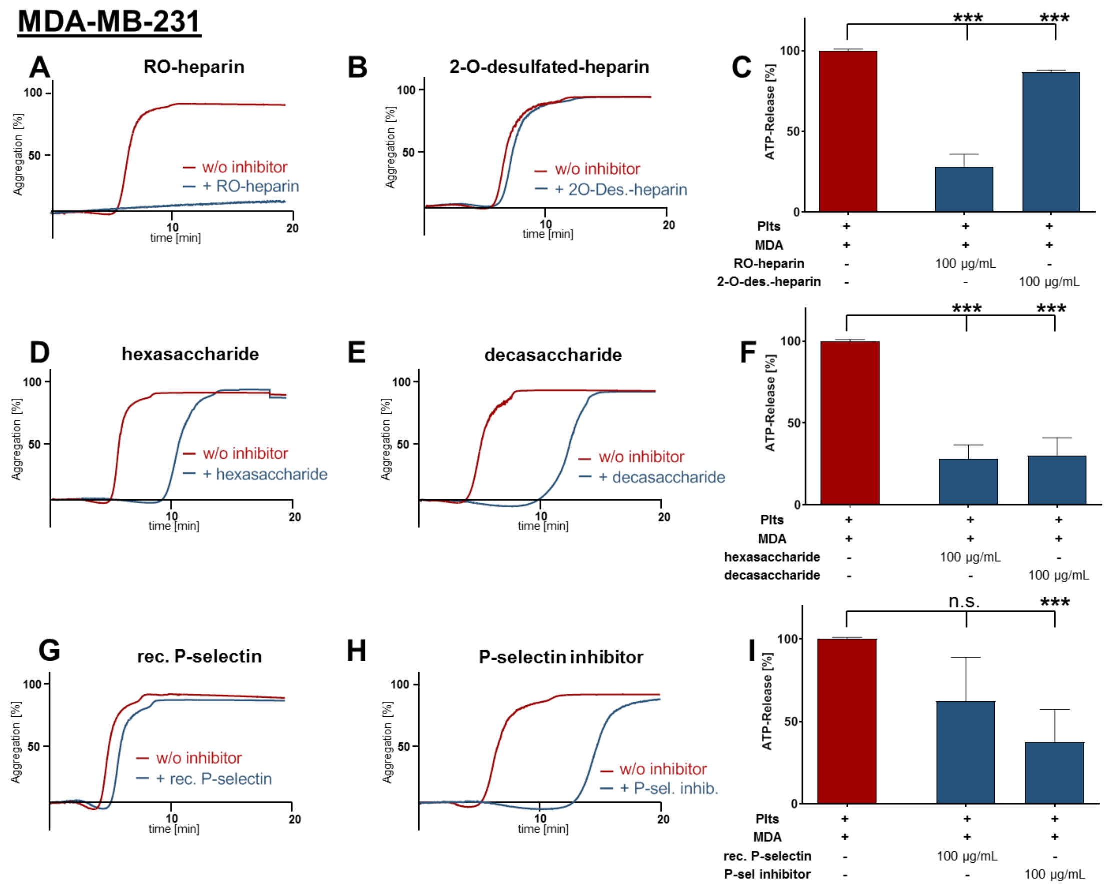

Figure S1. Impact of heparin derivatives on platelet aggregation and secretion. (**A**,**B**) Representative traces showing platelet-tumor cell aggregation in response to MDA-MB-231 cells. Platelets were preincubated with (**A**) 100 µg/mL RO-heparin or (**B**) 100 µg/mL 2-O-desulfated heparin. (**C**) Quantification of ATP release from MDA-MB-231 cell stimulated platelets preincubated with 100 µg/mL RO-heparin, or 100 µg/mL 2-O-desulfated heparin. (**D**,**E**) Representative traces showing platelet-tumor cell aggregation in response to MDA-MB-231 cells, platelets were preincubated with (**D**) 100 µg/mL hexasaccharide or (**E**) 100 µg/mL decasaccharide (n = 5). (**F**) Quantification of ATP release from MDA-MB-231 cell stimulated platelets preincubated with 100 µg/mL hexasaccharide, or 100 µg/mL decasaccharide. (**G**) Representative traces showing platelet-tumor cell aggregation in response to MDA-MB-231 cells. Tumor cells were preincubated with 1 µg/1000 cells recombinant human P-selectin (n = 5). (**H**) Representative traces showing platelet-tumor cell aggregation in response to MDA-MB-231 cells. Platelets were preincubated with 100 µg/mL P-selectin inhibitor (n = 5). (**I**) Quantification of ATP release from MDA-MB-231 cell (preincubated with 1 µg recombinant human P-selectin/1000 cells in some experiments) stimulated platelets preincubated with 100 µg/mL P-selectin inhibitor.
